# Eyes of Africa: The Genetics of Blindness: Study Design and Methodology

**DOI:** 10.1186/s12886-021-02029-8

**Published:** 2021-07-09

**Authors:** Olusola Olawoye, Chimdi Chuka-Okosa, Onoja Akpa, Tony Realini, Michael Hauser, Adeyinka Ashaye

**Affiliations:** 1 Department of Ophthalmology, College of Medicine, University of Ibadan, Ibadan, Nigeria; 2 Department of Ophthalmology, College of Medicine, University of Nigeria, Enugu Campus, Enugu, Nigeria; 3 Department of Epidemiology and Medical Statistics, College of Medicine, University of Ibadan, Ibadan, Nigeria; 4 Department of Ophthalmology and Visual Sciences, West Virginia University, Morgantown, USA; 5 Department of Medicine, Duke University, NC Durham, USA; 6 Department of Ophthalmology, Duke University, NC Durham, USA

**Keywords:** Glaucoma, Genetics, Africa, Methodology

## Abstract

**Background:**

This report describes the design and methodology of the “Eyes of Africa: The Genetics of Blindness,” a collaborative study funded through the Human Heredity and Health in Africa (H3Africa) program of the National Institute of Health.

**Methods:**

This is a case control study that is collecting a large well phenotyped data set among glaucoma patients and controls for a genome wide association study. (GWAS). Multiplex families segregating Mendelian forms of early-onset glaucoma will also be collected for exome sequencing.

**Discussion:**

A total of 4500 cases/controls have been recruited into the study at the end of the 3rd funded year of the study. All these participants have been appropriately phenotyped and blood samples have been received from these participants. Recent GWAS of POAG in African individuals demonstrated genome-wide significant association with the *APBB2* locus which is an association that is unique to individuals of African ancestry. This study will add to the existing knowledge and understanding of POAG in the African population.

**Supplementary Information:**

The online version contains supplementary material available at 10.1186/s12886-021-02029-8.

## Background

Glaucoma is a leading cause of irreversible blindness worldwide, and primary open angle glaucoma (POAG) is the most common subtype. POAG disproportionately affects people of African ancestry [1–5]. Prevalence in individuals over the age of 40 years is approximately 1 % in Europeans, compared with 4–5 % in African Americans [6], and 6.8 % in Ghana, West Africa [7]. Not only is POAG more common in African ancestry populations, but it also has an earlier onset [6,8] and a more rapid progression [2, 8]. Together, these factors result in a tremendous personal and societal burden of glaucomatous vision loss and blindness in Sub-Saharan Africa. Yet, despite the magnitude of this disease burden, we know little about what distinguishes the underlying genetic susceptibilities of glaucoma (and most other gene-based diseases) in Africans compared to other populations [9]. There is a lack of diversity in all large-scale genetic studies, most of which are performed in Caucasians [10]. This lack of diversity can arise from limited availability of research funding and limited research infrastructure in resource-poor environments, and from the reality that many under-represented study participants mistrust biomedical research given a history of exploitation [11, 12]. All of these factors apply to genetic studies of glaucoma in Africa: there is a critical shortage both of ophthalmologists specializing in glaucoma and the expensive diagnostic equipment required for such studies [13]. In addition, glaucoma is poorly understood by the patient population and the symptoms of the disease are difficult to recognize. Taken together, these factors have resulted in a huge gap in our knowledge of the genetics of glaucoma in Sub-Saharan African.

We have undertaken a study designed to address this knowledge gap: “Eyes of Africa: The Genetics of Blindness.” The goals of this study are to better understand the underlying genetic factors that lead to the higher prevalence and greater severity of glaucoma in Africans compared to other populations. The study is in three parts: a genome-wide association scan (GWAS); a sequence analysis of early onset Mendelian subtypes of glaucoma; and an educational outreach component to help inform community members about genetic research in general and glaucoma in particular, in order to facilitate future research and clinical management of glaucoma in Sub-Saharan Africa. In this paper, we describe the methodology for our quantitative genetic studies.

## Methods

### Study Organization

The Eyes of Africa study was funded as a Collaborative Network by the National Institutes of Health through the Human Heredity and Health in Africa (H3Africa) program (https://h3africa.org/). There are six collaborating clinical ascertainment centers across Sub Saharan Africa: three in Nigeria, and one each in Ghana, Malawi and South Africa. The collaborating institutions in Nigeria are the College of Medicine of the University of Ibadan, the College of Medicine University of Nigeria Nsukka, Enugu, and the Navy Hospital in Lagos. Other collaborating institutions are the Korle Bu Teaching Hospital at the University of Ghana in Accra, the Eyes of Africa clinic in Lilongwe, Malawi, and the Eye Center East London South Africa. Duke University plays an advisory role and assists with data analysis. The Principal Investigator of the study (AA) is located at the University of Ibadan, where she directs the study through the Administrative Core.

#### Ethical Considerations

The study protocol was reviewed and approved by the University of Ibadan/ University College Hospital Research Ethics Committee. Written informed consent was obtained from all participants before recruitment into the study.

### Participants

 Written consent to participate was obtained from all potential participants in their local language prior to screening and before any study-related interventions (including examination for eligibility). Eligible subjects who wished to participate were enrolled. The study used broad consent to enable use of the samples and associated clinical data in as many of the H3Africa-sponsored research projects as possible.

### Study Design

There are three components to the Eyes of Africa study: (1) A Genome Wide Association Study (GWAS) to identify new POAG susceptibility genes through collection and analysis of a large, well-phenotyped set of POAG cases and unaffected controls; (2) Sequence analysis of multiplex pedigrees to identify Mendelian forms of early onset glaucoma; and (3) A series of qualitative research studies to understand knowledge and attitudes about glaucoma, to educate and inform community members about glaucoma, and to conduct clinical screening of high-risk families and individuals. The design of the latter component (qualitative studies) will be reported separately.

#### GWAS

Participants in the GWAS study were recruited through regularly scheduled glaucoma clinics (cases) or general medicine clinics (controls) at the six sites listed above. In general, POAG cases were defined as all persons above the age of 40 years, with typical glaucomatous optic neuropathy characterized by cupping, pallor, thinning or loss of retinal nerve fiber layer on optic disc examination and/or with optical coherence tomography (OCT), with characteristic visual field (VF) loss and open angles on gonioscopy, and had no other ocular abnormality (secondary causes) to account for these changes. These criteria are consistent with the International Society of Geographical and Epidemiologic Ophthalmology (ISGEO) classification system [14]:

1. The highest level of evidence is eyes with optic disc abnormalities: vertical cup to disc ratio (VCDR) in the 97.5th percentile in the normal population and VF defects compatible with glaucoma, e.g. eyes with VCDR ≥ 0.7 and/or VCDR asymmetry ≥ 0.2 or a neuro-retinal rim width reduced to ≤ 0.1 CDR (between 11 and 1 o’clock or 5 to 7 o’clock) that also shows a definite visual field defect consistent with glaucoma.
2. A severely damaged optic disc (VCDR 99.5th percentile of the hyper-normal population) was sufficient to make the diagnosis if a VF test could not be performed satisfactorily (e.g. VCDR ≥ 0.9 in which a visual field could not be performed satisfactorily.)
3. In the presence of media opacity preventing optic disc exam and VF testing, then a visual acuity 20/400, plus any of the following: evidence of glaucoma filtering surgery, current use of anti-glaucoma medications or medical records showing a history of glaucoma-related vision loss.

All individuals with other forms of glaucoma apart from POAG were excluded from this study. This included the presence of any potential secondary causes of glaucoma, such as pigment dispersion, narrow angles, or previous ocular injury. All individuals with greater than 8 diopters of myopia or hyperopia were excluded from both cases and controls.

Controls were defined as individuals over the age of 40, with no history of IOP > 22mmHg, who were not affected by primary or secondary glaucoma.

#### Juvenile Open Angle Glaucoma (JOAG) Sequence Scan

Index cases and their family members participating in the JOAG study were recruited from the University of Ibadan site. All glaucoma cases younger than 40 years with all the above characteristic features of POAG were classified as Juvenile Open angle glaucoma (JOAG). Family history of vision difficulties was obtained, and if other family members were suspected of being glaucoma cases, a field visit to examine and recruit additional family members was undertaken.

### Study Assessments

Basic demographic information was collected, including ethnicity and native language of both participants and parents. General health information has been collected by all H3Africa-sponsored studies, including body mass index, blood pressure, medication history, smoking and alcohol use, and general medical history. All study participants underwent a comprehensive ocular examination. Slit lamp bio-microscopy was used to examine the conjunctiva, cornea, anterior chamber, iris, and lens. Automated refraction and keratometry (Auto Refractometer KR-800, Topcon Ltd, Tokyo, Japan) measurements, central corneal thickness, axial length and anterior chamber depth, lens diameter (PacScan plus:Sonomed model 300AP) measurements were performed. Using the results of the automated refractometer, best corrected distant visual acuity was determined for a distance of 6 m, and as near vision at a distance 25–30 cm using Jaeger charts, uncorrected and corrected using an addition for near vision. Perimetry was then performed using the Humphrey matrix visual field perimeter (Carl Zeiss Meditec, Inc., Dublin, CA), with the 24 − 2/10 − 2 SITA-standard program. Optical coherence tomography of the retinal nerve fiber layer was obtained using the Optovue machine (iscan Optovue OCT model: Ivue 500). The anterior chamber depth was evaluated and graded using Van Herrick’s system. Intraocular pressure (IOP) was measured with the Goldmann applanation tonometer.

 Gonioscopy was performed on all subjects using the Posner 4-mirror lens and the angles were graded according to the Shaffer’s grading system. Only patients with open angles were recruited into the study. All eyes with open angles were dilated after gonioscopy. The optic disc was examined using a handheld, 78-D lens and 10x eye piece of the slit lamp for stereoscopic evaluation of the vertical optic disc and cup diameters with an eyepiece micrometer scale. Notching of the disc rim (defined as partial or complete loss of neuro-retinal rim over one or more clock hours in the superior or inferior quadrants), or optic pits, disc drusen, and disc hemorrhage were noted.

A hard-copy clinical report form was used to record all data collected during subject examinations. As described below, the data are subsequently entered into a REDCap (Research Electronic Data Capture) database hosted at Duke University, Durham, North Carolina.

### Sample Management

#### Sample Collection

After the examination stage, blood samples were collected from eligible cases and controls by research assistants and medical laboratory scientists trained on the biorepository process of the project before commencement of study and sample collection. Kits for the sample collection were centrally procured by the Eyes of Africa administrative core and distributed to all the other centers. Prior to collecting the samples, strips of barcode labels were applied to the tubes, and to informed consent and case report forms. A 10 ml sample of venous blood was collected in EDTA tubes from cases and controls for DNA extraction and genetic analysis. This bottle was covered, sealed and placed in a small sized cooler containing ice packs. Blood samples were kept in these ice packs for a maximum of 2 h to maintain proper cold chain system during transit to the laboratory. The box was sealed and clearly marked biohazard material.

#### Sample Processing

Each transported sample was accompanied by relevant identification details and duly filled questionnaire. The laboratory was notified ahead of sample collection. Blood sample bottles collected were centrifuged and separated into different fractions (two plasma aliquots, one buffy coat, and one red cell concentrate, the latter of which contains small amounts of white cells and could be used as a backup source of DNA should extraction of the buffy coat fail). All four sample tubes were immediately frozen in barcoded cryo-vials in -80 C at the coordinating centers where ultra-low temp freezers are available. In collaboration with the H3 Africa biobank in Abuja [15], we developed a protocol for collection, shipping, and processing of blood samples to ensure an uninterrupted cold chain. This primarily involved the shipment of samples in credo boxes—Styrofoam-insulated cardboard boxes lined on all 6 sides with cold packs chilled to -80 C or -20 C prior to shipment. The Abuja biobank has demonstrated that this system enables overnight shipping without sample thaw [15].

At two sites within Nigeria (Enugu and Lagos) no -80 C storage was available to the study. Accordingly, our study protocol was modified. After blood centrifugation, sample aliquots were stored locally in -20 C for a maximum of 2 weeks and subsequently shipped in credo boxes to the central biorepository in Ibadan. Samples that arrived frozen were shifted to -80 C, while those that had thawed en-route were immediately extracted for DNA. Temperature of all the freezers (-20 degrees and − 80 degrees) were monitored daily with a temperature log. Batches of buffy coats stored at -80 C were shipped periodically from Ibadan to the Abuja Biobank for DNA extraction and sample quality control testing. DNA aliquots were shipped from Abuja to the core laboratories where downstream DNA testing was performed.

### Data Management

At the time of subject ascertainment, all study subject data were entered onto a paper case record form (CRF). Subsequently, a password protected and encrypted REDCap Electronic database was set up at Duke University and was used to archive the subject data. In order to ensure data accuracy and enhance data completeness, the database incorporated value range checks and logic checks to ensure mandatory fields were not skipped and data entered were appropriate for the field. The study Biostatistician performed intermittent real time data management by generating data quality control queries including valid ranges, consistency checking, completeness etc. Only staff listed in the delegation log were provided with unique individual password to access the REDCap platform for data entry from multiple study sites. Only the Biostatistician was provided with permission for data download, upload, editing and overall management.

#### Statistical Analysis Plan

 The primary analysis is designed to assess association between exposure variables (including central corneal thickness, mean keratometric readings, mean anterior chamber depth, mean lens diameter, optical coherence tomogram etc.) and case control status using generalized linear (regressions) models with binomial link function. Odds ratios (with their respective 95 % confidence intervals [CI]) will be estimated to test association between secondary exposure variables such as sociodemographic characteristics, hypertension status (or blood pressure), lifestyle factors (cigarette smoking, alcohol use etc.) and case-control status using logistic regression with and without adjustments for confounding factors. Adjusted population attributable risks (PARs) with their respective 95 % CIs for each exposure variable included in the best-fitted adjusted models will be calculated [16, 17]. The PARs will be estimated as the proportion of the risk of POAG in the population that is attributable to the individual risk factors.

#### Power/Sample Size Justification for the Genomic Data

We will utilize GWAS in a case-control study of 4000 POAG cases and 4000 controls. Based on reported single nucleotide polymorphisms (SNPs) associated with POAG, a sample size of at least 4000 case-control pairs provides > 90 % power to detect a SNP with genetic odds ratio of at least 1.2 and minor allele frequency of 0.2.

#### Power Justification for the Phenomic Data

A sample size of 8000 (4000 case and 4000 controls) provides substantial power to quantify the contributions of traditional and novel risk factors for Primary Open Angle Glaucoma (POAG). Assuming at least 5 % probability of exposure in the control population and a 0.75 correlation coefficient for exposure between cases and controls, a sample size of 4000 in each group will allow detecting an odds ratio of ≥ 1.0035 with a power of ≥ 80 % using logistic regression [18].

## Discussion

 There have been many large GWAS studies of POAG, and a recent multi-ethnic meta-analysis demonstrated association of 127 loci with genome-wide significance [19]. However, it remains unclear just how relevant these loci are to Africans and individuals of African ancestry around the world. The 3 GWAS analyses of POAG in African Americans published to date found modest association with many of the loci identified in Caucasians, yet failed to identify any replicated genome-wide significant results [20–22]. This is due at least in part to reduced statistical power arising from admixture in these populations, and population diversity within Africa raises similar challenges to genetic analysis [23]. At the same time, these findings likely reflect real differences in the genetic architecture of glaucoma in African populations. Indeed, the largest GWAS of POAG in African individuals to date demonstrated genome-wide significant association with the *APBB2* locus [24], an association that is unique to individuals of African ancestry.

POAG is not alone in this regard—other diseases also show distinct genetic architecture in different populations. These differences may arise from population bottlenecks and their effects on allele frequency. For example, the most common mutation causing cystic fibrosis in Europeans (ΔF508) is much less common in Africans, who have a correspondingly lower prevalence of cystic fibrosis [25]. Alternatively, they may arise through environmental influences. For example, the G1 and G2 alleles of apolipoprotein L1 (*APOL1*) are strongly associated with non-diabetic end-stage kidney disease (ESKD) in individuals of African ancestry [26]. These alleles are common in African populations, which partially explains the higher incidence of ESKD as compared with European populations [27]. These alleles likely arose as a result of positive selection because they confer resistance to infection by *Trypoanosoma brucei*, which causes African sleeping sickness [28].

 Regardless of the mechanisms by which they arise, such differences in disease etiology have profound consequences regarding both diagnosis and treatment of disease. Increasingly, DNA genotyping is being used to assist in the diagnosis of disease, either by gene sequencing to detect rare causative variants, or through the use of polygenic risk scores that combine the effects of many common genetic variants. Both of these approaches require extensive databases of genetic polymorphisms and mutations that are specific to the population in which they are being used. Such databases have been constructed through analysis of primarily European populations, and do not work well in populations of African ancestry, leading to missed diagnoses and incomplete assessments of disease risk. Similarly, treatments based on disease mechanisms learned through analysis of other populations will be ineffective when applied to Africans. Such problems will only increase as clinical care moves more towards personalized medicine.

The impact of this group of studies is expected to be multi-fold. Phenotypic data will characterize the nature of the clinical glaucoma burden in SSA. Glaucoma treatment resources are greatly limited in SSA, and any programmatic effort to expand therapeutic capacity will depend on a clear understanding of the burden of clinical disease in order to successfully address region-specific needs. Genotypic data will provide insight into the complex and incompletely-characterized pathophysiology of glaucoma. Such information can demonstrate mechanisms and pathways active in the glaucoma disease process, reveal unrecognized factors contributing to the development and progression of disease, elucidate novel therapeutic targets, among many other valuable lessons. The sequence scan in patients with early-onset JOAG may enhance our understanding of the factors that lead some people to develop glaucoma at an early age and may clarify factors upon which more effective screening programs can be developed. Overall, the Eyes of Africa study is intended to provide a better understanding of the genetic basis of glaucoma in SSA with the goal of reducing preventable glaucoma-related vision loss and blindness.

 We have encountered multiple challenges in the conduct of this study. The COVID-19 pandemic interrupted and subsequently slowed all aspects of the study. Recruitment of study subjects has been hampered because genetics in general is not well understood by the general population, making many potential study subjects reluctant or fearful to participate. The clinical presentation of POAG makes this even more difficult, as glaucoma cases may not be aware they have the disease and are thus less motivated to join the study. Recruitment of appropriate controls is also difficult, as they should be as old as possible and also devoid of any features suggestive of glaucoma. In order to mitigate these difficulties, we have organized community outreaches and educational programs. These programs will be described in more detail separately, but in brief, we have established a community advisory board made up of public health leaders, key opinion leaders, community leaders, and glaucoma patients association at the University College Hospital. The board members meet regularly with the community engagement team, which is made up of social scientists, community entry personnel, and community ophthalmologists. These two teams collaborate to educate the community members and ensure referral of other family members to the hospital for screening and diagnosis. The primary objective of our community engagement program is to provide information about genetic studies in general, as well as our glaucoma study in particular. It is especially important to educate community members about the differences between blinding ocular conditions such as cataracts, in which blindness can be reversed through a simple operation, as compared to glaucoma, where blindness is irreversible but progression can be slowed through treatment.

Other challenges with this study pertain to the maintenance of equipment. We have encountered breakdowns in the Optical Coherence TOmography (OCT) machine and the Humphrey matrix visual field units purchased for this study, and field repair of such units is difficult in the African setting, so we were unable to use these diagnostic approaches for some research subjects. Consequently, in such situations when visual fields and/or OCTs could not be obtained, only participants who fit the diagnostic criteria 2 and 3 of the ISGEO classification were recruited and included in the study. Electricity to power the freezers at different sites is a serious problem—power outages are a daily occurrence in many parts of Nigeria. This issue necessitates the use of alternative power arrangements for the − 80 C freezer purchased for the Ibadan site. This freezer is supported by a large solar array which charges a bank of lead-acid batteries. Low-voltage DC current from this battery bank is converted to high-voltage AC current with an inverter and step-up transformer. While this system prevents damage to the freezer compressor from frequent power outages, it is accompanied by a four-fold increase in cost.

Despite all of these difficulties, within the first 3 years of our funding, we have collected more than 4500 blood samples, which are currently being extracted for DNA preparatory to genotyping on the Illumina H3Av2 BeadChip. With COVID-19 restrictions being lifted, we hope to come close to achieving our clinical ascertainment goals of 8000 total samples. We hope that the genotyping of these samples and their subsequent genetic analysis will continue to expand our understanding of the genetic architecture of primary open angle glaucoma in Sub-Saharan Africa. This better understanding of the molecular etiology of glaucoma will improve both the diagnosis and treatment of this blinding disease, reducing the tremendous burden of human suffering that it causes, both in Africa and in populations of the African diaspora around the world.

## Supplementary Information

**Additional file 1.**

## Data Availability

In accordance with H3Africa guidelines, clinical and genetic data will be made available through the H3Africa Bioinformatics Network.

## Abbreviations

POAG: Primary open angle glaucoma
GWAS: Genome-wide association study
IOP: Intra-ocular pressure
VCDR: Vertical cup to disc ratio
JOAG: Juvenile open angle glaucoma
