# Supplementary material for: Eyes of Africa: The Genetics of Blindness: Study Design and Methodology
Source: BMC Ophthalmol. 2021 Jul 9;21:272. doi: 10.1186/s12886-021-02029-8 (PMC8267233; doi:10.1186/s12886-021-02029-8)
Supplement: Supplementary file 1 — Additional file 1. [file 12886_2021_2029_MOESM1_ESM.pdf]

# The Genetics of Blindness

| | |
|---|---|
| Record ID | ________________ |
| Type of Case | ◯ Case ◯ Control |
| Date of Examination | ________________ |
| Identifier Code | ________________ |
| Specimen Number | ________________ |
| Case Note Number | ________________ |
| Collection of Sample Date | ________________ |
| What is your birth date? | ◯ Enter Birth Date ◯ Don't Know ◯ Prefer Not to Answer |
| MM/DD/YYYY | ________________ |
| About how old are you? | ◯ Enter age ◯ Don't Know ◯ Prefer Not to Answer |
| Age | ________________ (Years) |
| Are you male or female? | ◯ Male ◯ Female ◯ Prefer Not to Answer ◯ Don't Know |

| | No formal education | Quranic education | Primary education | Secondary education | Tertiary education | Other |
|---|---|---|---|---|---|---|
| Educational Level | ◯ | ◯ | ◯ | ◯ | ◯ | ◯ |

| | |
|---|---|
| Other education level | ________________ |
| Occupational status | ◯ Employed ◯ Unemployed |
| What is your occupation? | ________________ |
| Domicile | ________________ |

**In which country were you and your parents born?**

| | Nigeria | Ghana | Kenya | South Africa | Other African Country | Other non-African Country |
|---|---|---|---|---|---|---|
| You | ○ | ○ | ○ | ○ | ○ | ○ |
| Your Mother | ○ | ○ | ○ | ○ | ○ | ○ |
| Your Father | ○ | ○ | ○ | ○ | ○ | ○ |

In which African country were you born? __________________________________

In which non-African country were you born? __________________________________

In which African country was your mother born? __________________________________

In which non-African country was your mother born? __________________________________

In which African country was your father born? __________________________________

In which non-African country was your father born? __________________________________

**What is the main language spoken by you and your parents?**

| | Yoruba | Igbo | Hausa | Efik | Itsekiri | Fulani | Calabar | Other |
|---|---|---|---|---|---|---|---|---|
| You | ○ | ○ | ○ | ○ | ○ | ○ | ○ | ○ |
| Your Mother | ○ | ○ | ○ | ○ | ○ | ○ | ○ | ○ |
| Your Father | ○ | ○ | ○ | ○ | ○ | ○ | ○ | ○ |

What is the main language spoken by you? __________________________________

What is the main language spoken by your mother? __________________________________

What is the main language spoken by your father? __________________________________

**What is your and your parent's ethnic or tribal affiliation?**

| | Itsekiri | Idoma | Fulani | Yoruba | Calaba | Urhobo | Hausa | Ibibio | Igbo | Other |
|---|---|---|---|---|---|---|---|---|---|---|
| You | ○ | ○ | ○ | ○ | ○ | ○ | ○ | ○ | ○ | ○ |
| Your Mother | ○ | ○ | ○ | ○ | ○ | ○ | ○ | ○ | ○ | ○ |
| Your Father | ○ | ○ | ○ | ○ | ○ | ○ | ○ | ○ | ○ | ○ |

What is your ethnic or tribal affiliation? __________________________________

What is your mother's ethnic or tribal affiliation? __________________________________

What is your father's ethnic or tribal affiliation? __________________________________

## Standing Height

What is your standing height? Please select appropriate units and measure your height two times. ◯ Inches ◯ Centimeters

Standing Height 1 __________________________________ (Inches)

Standing Height 2 __________________________________ (Inches)

Standing Height 3 __________________________________ (Inches)

Standing Height Average __________________________________ (Inches)

Standing Height 1 __________________________________ (Centimeters)

Standing Height 2 __________________________________ (Centimeters)

Standing Height 3 __________________________________ (Centimeters)

Standing Height Average __________________________________ (Centimeters)

## Measured Weight

What is your weight? Please select the appropriate units and measure your weight twice. ◯ lbs ◯ kg

Are you wearing a cast or medical prosthesis? ◯ Yes ◯ No

Where is your cast or medical prosthesis located? __________________________________

Are you wearing street clothes? ◯ Yes ◯ No

Measured Weight 1

__________ (lbs)

Measured Weight 2

__________ (lbs)

Measured Weight 3

__________ (lbs)

Average Measured Weight

__________ (lbs)

Measured Weight 1

__________ (kg)

Measured Weight 2

__________ (kg)

Measured Weight 3

__________ (kg)

Average Measured Weight

__________ (kg)

## Blood Pressure

Has a doctor ever said that you have high blood pressure or hypertension?

◯ Yes ◯ No ◯ Not Sure

At what age were you first told this?

__________

Was this during pregnancy only?

__________

Have you ever taken medication for hypertension/high blood pressure?

◯ Yes ◯ No ◯ Not Sure

At what age did you begin taking medicine for this?

◯ Age ◯ Not Sure

Age

__________

Are currently taking medication for hypertension/high blood pressure?

◯ Yes ◯ No

| | |
|---|---|
| Aneroid Sphygmomanometers Name | __________________ |
| Aneroid Sphygmomanometers Model | __________________ |
| Blood pressure cuffs size | ○ S ○ M ○ L ○ XL |
| Systolic Pressure Measurement 1 | __________________ |
| Diastolic Pressure Measurement 1 | __________________ |
| Systolic Pressure Measurement 2 | __________________ |
| Diastolic Pressure Measurement 2 | __________________ |
| Systolic Pressure Measurement 3 | __________________ |
| Diastolic Pressure Measurement 3 | __________________ |
| Date of Measurement(s) | __________________ |

## Tobacco

| | |
|---|---|
| Have you smoked at least 100 cigarettes in your entire life? | ○ Yes ○ No ○ Don't Know |

| | Every Day | Some Days | Not at All | Don't Know | Prefer Not to Answer |
|---|---|---|---|---|---|
| How often do you smoke? | ○ | ○ | ○ | ○ | ○ |

| | |
|---|---|
| Have you EVER smoked cigarettes EVERY DAY for at least 6 months? | ○ Yes ○ No ○ Don't Know |
| How old were you when you first started smoking cigarettes every day? | ○ Enter age ○ Don't Know |
| Age | __________________ |
| How old were you when you first started smoking cigarettes FAIRLY REGULARLY? | ○ Enter age ○ Don't Know |
| Age | __________________ |

| | |
|---|---|
| On the average, about how many cigarettes do you now smoke each day? | ◯ Enter number ◯ Don't Know ◯ Prefer Not to Answer |
| Number of cigarettes per day | __________________ |
| On how many of the past 30 days did you smoke cigarettes? | ◯ Enter number ◯ Don't Know ◯ Prefer Not to Answer |
| Number of days | __________________ |
| On the average, on the days you smoked, how many cigarettes did you usually smoke each day? | ◯ Enter number ◯ Don't Know ◯ Prefer Not to Answer |
| Number of cigarettes smoked per day | __________________ |
| Have you EVER smoked cigarettes EVERY DAY for at least 6 months? | ◯ Yes ◯ No ◯ Don't Know ◯ Prefer Not to Answer |
| When you last smoked every day, on average how many cigarettes did you smoke each day? | ◯ Enter number ◯ Don't Know ◯ Prefer Not to Answer |
| Number of cigarettes per day | __________________ |
| When you last smoked fairly regularly, on average how many cigarettes did you smoke each day? | ◯ Enter number ◯ Don't Know ◯ Prefer Not to Answer |
| Number of cigarettes per day | __________________ |

| | Enter days | Enter weeks | Enter months | Enter years | Don't Know | Prefer Not to Answer |
|---|---|---|---|---|---|---|
| About how long has it been since you COMPLETELY quit smoking cigarettes? | ◯ | ◯ | ◯ | ◯ | ◯ | ◯ |

| | |
|---|---|
| Days | __________________ |
| Weeks | __________________ |
| Months | __________________ |
| Years | __________________ |

## In your ENTIRE LIFE, have you ever

| | |
|---|---|
| Smoked at least 50 cigars? | ◯ Yes ◯ No ◯ Don't Know ◯ Prefer Not to Answer |
| Smoked a pipe at least 50 times? | ◯ Yes ◯ No ◯ Don't Know ◯ Prefer Not to Answer |
| Used snuff, [such as Skoal?, Skoal Bandit? or Copenhagen?] at least 20 times? | ◯ Yes ◯ No ◯ Don't Know ◯ Prefer Not to Answer |
| Used chewing tobacco, [such as Redman?, Levi Garrett? or Beechnut?] at least 20 times? | ◯ Yes ◯ No ◯ Don't Know ◯ Prefer Not to Answer |

## Alcohol

| | |
|---|---|
| In your entire life, have you had at least 1 drink of any kind of alcohol, not counting small tastes or sips? | ◯ Yes ◯ No ◯ Don't Know ◯ Prefer Not to Answer |
| About how old were you when you first started drinking, not counting small tastes or sips of alcohol? | ◯ Age ◯ Never drank |
| Age | ______________________ |
| During the past 30 days, on how many days did you drink one or more drinks of an alcoholic beverage? | ◯ Enter number of days ◯ Don't Know ◯ Prefer Not to Answer |
| Number of days | ______________________ |
| On the days that you drank during the past 30 days, how many drinks did you usually have each day? Count as a drink a can or bottle of beer; a wine cooler or a glass of wine, champagne, or sherry; a shot of liquor or a mixed drink or cocktail. | ◯ Enter number of drinks ◯ Don't Know ◯ Prefer Not to Answer |
| Number of drinks | ______________________ |
| What was the LARGEST number of drinks that you ever drank in a single day? | ◯ Enter number ◯ Don't Know ◯ Prefer Not to Answer |
| Largest number of drinks in a single day | ______________________ |

| Have you ever used any of the following? | |
|---|---|
| Sedatives | ◯ Yes ◯ No ◯ Don't Know |
| Age at first use | ◯ Enter Age ◯ Don't Know |
| Age | __________________ |
| How many days in the last 30 days did you use sedatives? | ◯ Enter number of days<br>◯ Don't Know |
| Number of days | __________________ |
| Tranquilizers | ◯ Yes ◯ No ◯ Don't Know |
| Age at first use | ◯ Enter Age ◯ Don't Know |
| Age | __________________ |
| How many days in the last 30 days did you use tranquilizers? | ◯ Enter number of days<br>◯ Don't Know |
| Number of days | __________________ |
| Painkillers | ◯ Yes ◯ No ◯ Don't Know |
| Age at first use | ◯ Enter Age ◯ Don't Know |
| Age | __________________ |
| How many days in the last 30 days did you use painkillers? | ◯ Enter number of days<br>◯ Don't Know |
| Number of days | __________________ |
| Stimulants | ◯ Yes ◯ No ◯ Don't Know |
| Age at first use | ◯ Enter Age ◯ Don't Know |
| Age | __________________ |
| How many days in the last 30 days did you use stimulants? | ◯ Enter number of days<br>◯ Don't Know |
| Number of days | __________________ |

| | |
|---|---|
| Marijuana, Hash, HC, or Grass | ◯ Yes ◯ No ◯ Don't Know |
| Age at first use | ◯ Enter Age ◯ Don't Know |
| Age | __________________ |
| How many days in the last 30 days did you use marijuana, hash, HC, or grass? | ◯ Enter number of days<br>◯ Don't Know |
| Number of days | __________________ |
| Cocaine | ◯ Yes ◯ No ◯ Don't Know |
| Age at first use | ◯ Enter Age ◯ Don't Know |
| Age | __________________ |
| How many days in the last 30 days did you use cocaine? | ◯ Enter number of days<br>◯ Don't Know |
| Number of days | __________________ |
| Crack Cocaine | ◯ Yes ◯ No ◯ Don't Know |
| Age at first use | ◯ Enter Age ◯ Don't Know |
| Age | __________________ |
| How many days in the last 30 days did you use crack cocaine? | ◯ Enter number of days<br>◯ Don't Know |
| Number of days | __________________ |
| Hallucinogens | ◯ Yes ◯ No ◯ Don't Know |
| Age at first use | ◯ Enter Age ◯ Don't Know |
| Age | __________________ |
| How many days in the last 30 days did you use hallucinogens? | ◯ Enter number of days<br>◯ Don't Know |
| Number of days | __________________ |

| | |
|---|---|
| Inhalants | ◯ Yes ◯ No ◯ Don't Know |
| Age at first use | ◯ Enter Age ◯ Don't Know |
| Age | ______________________ |
| How many days in the last 30 days did you use inhalants? | ◯ Enter number of days<br>◯ Don't Know |
| Number of days | ______________________ |
| Heroin | ◯ Yes ◯ No ◯ Don't Know |
| Age at first use | ◯ Enter Age ◯ Don't Know |
| Age | ______________________ |
| How many days in the last 30 days did you use heroin? | ◯ Enter number of days<br>◯ Don't Know |
| Number of days | ______________________ |
| Any other medicines, drugs, or substances? | ◯ Yes ◯ No ◯ Don't Know |
| Name of other medicine, drug, or substance | ______________________ |
| Age at first use | ◯ Enter Age ◯ Don't Know |
| Age | ______________________ |
| How many days in the last 30 days did you use this medicine, drug, or substance? | ◯ Enter number of days<br>◯ Don't Know |
| Number of days | ______________________ |

**Has a doctor ever told you that you had any of the following conditions?**

| | Yes | No | Don't Know | Refuse to Answer |
|---|---|---|---|---|
| High blood pressure | ◯ | ◯ | ◯ | ◯ |

| | |
|---|---|
| At what age were you told that you have high blood pressure? | ______________________ |

| | Yes | No | Don't Know | Refuse to Answer |
|---|---|---|---|---|
| High cholesterol (high fat in your blood) | ◯ | ◯ | ◯ | ◯ |

At what age were you told that you have high cholesterol (high fat in your blood)? ______________________

| | Yes | No | Don't Know | Refuse to Answer |
|---|---|---|---|---|
| Heart attack | ◯ | ◯ | ◯ | ◯ |

At what age were you told that you have had a heart attack? ______________________

| | Yes | No | Don't Know | Refuse to Answer |
|---|---|---|---|---|
| Arrhythmia (irregular heart beats) | ◯ | ◯ | ◯ | ◯ |

At what age were you told that you have an arrhythmia (irregular heart beats)? ______________________

| | Yes | No | Don't Know | Refuse to Answer |
|---|---|---|---|---|
| Asthma or wheezing | ◯ | ◯ | ◯ | ◯ |

At what age were you told that you have asthma or wheezing? ______________________

| | Yes | No | Don't Know | Refuse to Answer |
|---|---|---|---|---|
| Stroke | ◯ | ◯ | ◯ | ◯ |

At what age were you told that you have had a stroke? ______________________

| | Yes | No | Don't Know | Refuse to Answer |
|---|---|---|---|---|
| Kidney disease | ◯ | ◯ | ◯ | ◯ |

At what age were you told that you have kidney disease? ______________________

| | Yes | No | Don't Know | Refuse to Answer |
|---|---|---|---|---|
| Diabetes/High blood sugar | ◯ | ◯ | ◯ | ◯ |

At what age were you told that you have diabetes or high blood sugar? ______________________

| | Yes | No | Don't Know | Refuse to Answer |
|---|---|---|---|---|
| Cancer | ◯ | ◯ | ◯ | ◯ |

At what age were you told that you have cancer? ______________________

Where did the cancer start? ______________________

| | Yes | No | Don't Know | Refuse to Answer |
|---|---|---|---|---|
| Schizophrenia | ◯ | ◯ | ◯ | ◯ |

At what age were you told that you have schizophrenia? ____________________________

| | Yes | No | Don't Know | Refuse to Answer |
|---|---|---|---|---|
| Tuberculosis (TB) | ◯ | ◯ | ◯ | ◯ |

At what age were you told that you have tuberculosis (TB)? ____________________________

| | Yes | No | Don't Know | Refuse to Answer |
|---|---|---|---|---|
| Sleeping sickness | ◯ | ◯ | ◯ | ◯ |

At what age were you told that you have sleeping sickness? ____________________________

| | Yes | No | Don't Know | Refuse to Answer |
|---|---|---|---|---|
| AIDS (HIV) | ◯ | ◯ | ◯ | ◯ |

At what age were you told that you have AIDS (HIV)? ____________________________

| | Yes, now | Yes, not now | No | Maybe | Don't Know |
|---|---|---|---|---|---|
| Are you taking any anticoagulants (Coumadin, Warfarin, etc.)? | ◯ | ◯ | ◯ | ◯ | ◯ |

## History of Stroke - Ischemic Infarction and Hemorrhage

Were you ever told by a doctor you had a stroke? ◯ Yes ◯ No ◯ Don't Know

Were you ever told by a doctor you had a TIA, ministroke, or transient ischemic attack? ◯ Yes ◯ No ◯ Don't Know

Have you ever had a sudden painless weakness on one side of your body? ◯ Yes ◯ No ◯ Don't Know

Have you ever had a sudden numbness or a dead feeling on one side of your body? ◯ Yes ◯ No ◯ Don't Know

Have you ever had a sudden painless loss of vision in one or both eyes? ◯ Yes ◯ No ◯ Don't Know

Have you ever suddenly lost one half of your vision? ◯ Yes ◯ No ◯ Don't Know

Have you ever suddenly lost the ability to understand what people are saying? ◯ Yes ◯ No ◯ Don't Know

Have you ever suddenly lost the ability to express yourself verbally or in writing? ◯ Yes ◯ No ◯ Don't Know

## Personal History of Kidney Failure

Has a doctor ever told you that you had kidney failure? ◯ Yes ◯ No ◯ Don't Know

Are one or both kidneys working well now? ◯ Yes ◯ No ◯ Don't Know

How old were you when you were first told by a doctor that you had kidney failure? Indicate the actual age. ______________________

Are you currently on renal dialysis? ◯ Yes ◯ No ◯ Don't Know

Have you ever had a kidney transplant? ◯ Yes ◯ No ◯ Don't Know

Has anyone in your family had kidney disease or died from kidney disease (not a urinary tract infection)? ◯ Yes ◯ No ◯ Don't Know

Do you know the type of kidney disease? ◯ Yes ◯ No

What type of kidney disease did your family member have? ______________________

Has a doctor told you your kidneys have low function? ◯ Yes ◯ No ◯ Don't Know

Has a doctor told you your kidneys have low function? ◯ Yes ◯ No ◯ Don't Know

Have you ever had a urine test? ◯ Yes ◯ No ◯ Don't Know

When did you have a urine test? ______________________

Where did you have a urine test? ______________________

Do you know the doctor's name that performed the urine test? ◯ Yes ◯ No ◯ Don't Know

Please enter the name of the Doctor ______________________

| | Phone Number | Email | Address | Don't Know | Can't Remember |
|---|---|---|---|---|---|
| Please enter the contact information for the doctor | ☐ | ☐ | ☐ | ☐ | ☐ |

Doctor's Phone Number ______________________

Doctor's Email ______________________

Doctor's Address

Has your doctor or health care professional told you that you had diabetes? ◯ Yes ◯ No ◯ Don't Know

Are you taking medicine for this? ◯ Yes ◯ No ◯ Don't Know

What kind of medicine are you taking? ◯ Insulin ◯ Pills

At what age was this first treated? ◯ Enter age ◯ Don't Know

Enter age ______________________

Was insulin your first diabetes medicine? ◯ Yes ◯ No ◯ Don't Know

Did diabetes occur ONLY during Pregnancy? ◯ Yes ◯ No ◯ Don't Know

## Self-report of Human Immunodeficiency Virus Testing

Have you ever been tested for HIV? ◯ Yes ◯ No ◯ Don't Know

When did you have your most recent HIV test?

Month
◯ Unknown
◯ January
◯ February
◯ March
◯ April
◯ May
◯ June
◯ July
◯ August
◯ September
◯ October
◯ November
◯ December

Year ______________________

| | Negative | Positive | Never obtained results | Indeterminate | Prefer not to answer | Don't know |
|---|---|---|---|---|---|---|
| What was the result of your most recent HIV test? | ◯ | ◯ | ◯ | ◯ | ◯ | ◯ |

Is hypertension in your family history? ◯ Yes ◯ No ◯ Don't Know

Is stroke in your family history? ◯ Yes ◯ No ◯ Don't Know

Are you attending any of the clinics in UCH? ◯ Yes ◯ No

Which clinic are you attending? ______________________

Are you attending any other clinic outside of UCH? ◯ Yes ◯ No

Which non-UCH clinic are you attending? ______________________

Family history of glaucoma? ◯ Yes ◯ No

Who?
◯ Mother ◯ Father ◯ Brother
◯ Sister ◯ Cousin ◯ Aunt
◯ Uncle ◯ Niece ◯ Nephew
◯ Grandmother ◯ Grandfather
◯ Other

Other Family Member ______________________

## Visual Acuity

Unaided - Right Eye ______________________

Unaided - Left Eye ______________________

Best Corrected - Right Eye ______________________

Best Corrected - Left Eye ______________________

## Auto-Refraction

| | Seen | Not Seen |
|---|---|---|
| Target - Right Eye | ◯ | ◯ |
| Target - Left Eye | ◯ | ◯ |

sph - Right Eye ________________________________

sph - Left Eye ________________________________

cyl - Right Eye ________________________________

cyl - Left Eye ________________________________

axis - Right Eye ________________________________

axis - Left Eye ________________________________

What is responsible for poor/reduced vision in the Right Eye? ◯ Glaucoma ◯ Other

| | Cataract | Uveitis | Age related Macular degeneration | Diabetic retinopathy | Other |
|---|---|---|---|---|---|
| Other condition responsible for poor/reduced vision in the Right Eye | ◯ | ◯ | ◯ | ◯ | ◯ |

Specify Other ________________________________

What is responsible for poor/reduced vision in the Left Eye? ◯ Glaucoma ◯ Other

| | Cataract | Uveitis | Age related Macular degeneration | Diabetic retinopathy | Other |
|---|---|---|---|---|---|
| Other condition responsible for poor/reduced vision in the Left Eye | ◯ | ◯ | ◯ | ◯ | ◯ |

Specify Other ________________________________

## IOP as of now

| | Possible | Not Possible |
|---|---|---|
| Right Eye | ◯ | ◯ |
| Left Eye | ◯ | ◯ |

IOP 1 - Right Eye

__________________ (mmHG)

IOP 1 - Left Eye

__________________ (mmHG)

Date - Right Eye

__________________

Date - Left Eye

__________________

IOP 2 -Right Eye

__________________ (mmHG)

IOP 2 - Left Eye

__________________ (mmHG)

Date - Right Eye

__________________

Date - Left Eye

__________________

Highest IOP ever recorded - Right Eye

__________________ (mmHG)

Date - Right Eye

__________________

Highest IOP ever recorded - Left Eye

__________________ (mmHG)

Date - Left Eye

__________________

With medication?

◯ Yes
◯ No

## Vertical Cup to Disc Ratio

## Fundoscopy

| | Possible | Not Possible |
|---|---|---|
| Right Eye | ○ | ○ |
| Left Eye | ○ | ○ |

Vertical Cup to Disc Ratio - Right Eye

__________________________________

Vertical Cup to Disc Ratio - Left Eye

__________________________________

## Horizontal Cup to Disc Diameter

Horizontal Cup to Disc Diameter - Right Eye

__________________________________

Horizontal Cup to Disc Diameter - Left Eye

__________________________________

## Gonioscopy

| | Open angle | Closed angle | Narrow angle |
|---|---|---|---|
| Right Eye | ○ | ○ | ○ |
| Left Eye | ○ | ○ | ○ |

| | Pigments in Angles | Synechiae | Blood in Schlemms Canal | Other | None |
|---|---|---|---|---|---|
| Other findings on gonioscopy - Right Eye | ○ | ○ | ○ | ○ | ○ |

Other

__________________________________

| | Pigments in Angles | Synechiae | Blood in Schlemms Canal | Other | None |
|---|---|---|---|---|---|
| Other findings on gonioscopy -Left Eye | ○ | ○ | ○ | ○ | ○ |

Other

__________________________________

Diagnosis - Right Eye ______________________

Diagnosis - Left Eye ______________________

## Visiual Field

Target - Right Eye ◯ Seen ◯ Not Seen

What was used? - Right Eye ◯ CVF 24-2 ◯ CVF 10-2

Stimulus - Right Eye ◯ III ◯ V

MD - Right Eye ______________________

PSD - Right Eye ______________________

| | Outside normal limits | Within normal limits | Borderline | Generalized depression of sensitivities | Abnormally high sensitivity |
|---|---|---|---|---|---|
| GHT - Right Eye | ◯ | ◯ | ◯ | ◯ | ◯ |

Target - Left Eye ◯ Seen ◯ Not Seen

What was used? - Left Eye ◯ CVF 24-2 ◯ CVF 10-2

Stimulus - Left Eye ◯ III ◯ V

MD - Left Eye ______________________

PSD - Left Eye ______________________

| | Outside normal limits | Within normal limits | Borderline | Generalized depression of sensitivities | Abnormally high sensitivity |
|---|---|---|---|---|---|
| GHT - Left Eye | ◯ | ◯ | ◯ | ◯ | ◯ |

Type of treatment - Right Eye ◯ Medical ◯ Surgical ◯ Both ◯ No Treatment

List drugs

What type of surgery was done? ______________________

When was it done? ____________________

Type of treatment - Left Eye ◯ Medical ◯ Surgical ◯ Both ◯ No Treatment

List drugs

What type of surgery was done? ____________________

When was it done? ____________________

Samples collected ◯ Yes ◯ No

## Keratometric Reading

| | Possible | Not Possible |
|---|---|---|
| Right Eye | ◯ | ◯ |
| Left Eye | ◯ | ◯ |

Keratometric 1 - Right Eye ____________________

Keratometric 1 - Left Eye ____________________

Keratometric 2 - Right Eye ____________________

Keratometric 2 - Left Eye ____________________

Axial length - Right Eye ____________________
(mm)

Axial length - Left Eye ____________________
(mm)

Anterior Chamber Depth - Right Eye ____________________

Anterior Chamber Depth - Left Eye ____________________

Lens Diameter - Right Eye ____________________

Lens Diameter - Left Eye ______________________

Central Corneal Thickness - Right Eye ______________________

Central Corneal Thickness - Left Eye ______________________

## OCT

| | Possible | Not Possible |
|---|---|---|
| Right Eye | ○ | ○ |
| Left Eye | ○ | ○ |

Horizontal CDR on OCT - Right Eye ______________________

Vertical CDR on OCT - Right Eye ______________________

OCT Date - Right Eye ______________________

Horizontal CDR on OCT - Left Eye ______________________

Vertical CDR on OCT - Left Eye ______________________

OCT Date - Left Eye ______________________

Superior RNFL - Right Eye ______________________

Superior RNFL - Right Eye
- ○ Within normal limit
- ○ Borderline
- ○ Outside normal limit

Superior RNFL - Left Eye ______________________

Superior RNFL - Left Eye
- ○ Within normal limit
- ○ Borderline
- ○ Outside normal limit

Inferior RNFL - Right Eye ______________________

Inferior RNFL - Right Eye
- ○ Within normal limit
- ○ Borderline
- ○ Outside normal limit

| | |
|---|---|
| Inferior RNFL - Left Eye | __________________________________ |
| Inferior RNFL - Left Eye | ◯ Within normal limit<br>◯ Borderline<br>◯ Outside normal limit |
| Nasal RNFL - Right Eye | __________________________________ |
| Nasal RNFL - Right Eye | ◯ Within normal limit<br>◯ Borderline<br>◯ Outside normal limit |
| Nasal RNFL - Left Eye | __________________________________ |
| Nasal RNFL - Left Eye | ◯ Within normal limit<br>◯ Borderline<br>◯ Outside normal limit |
| Temporal RNFL - Right Eye | __________________________________ |
| Temporal RNFL - Right Eye | ◯ Within normal limit<br>◯ Borderline<br>◯ Outside normal limit |
| Temporal RNFL - Left Eye | __________________________________ |
| Temporal RNFL - Left Eye | ◯ Within normal limit<br>◯ Borderline<br>◯ Outside normal limit |
| Mean RNFL - Right Eye | __________________________________<br>(micrometers) |
| Mean RNFL - Right Eye | ◯ Within normal limit<br>◯ Borderline<br>◯ Outside normal limit |
| Mean RNFL - Left Eye | __________________________________<br>(micrometers) |
| Mean RNFL - Left Eye | ◯ Within normal limit<br>◯ Borderline<br>◯ Outside normal limit |
| Disc Diameter - Right Eye | __________________________________ |
| Disc Diameter - Left Eye | __________________________________ |

Rim Symmetry - Right Eye ________________

Rim Symmetry - Left Eye ________________

Rim Area - Right Eye ________________

Rim Area - Left Eye ________________

Disc Area - Right Eye ________________

Disc Area - Left Eye ________________

Cup Volume - Right Eye ________________

Cup Volume - Left Eye ________________

ONH Analysis - Right Eye ________________

ONH Analysis - Left Eye ________________

Area CD - Right Eye ________________

Area CD - Left Eye ________________

Vertical CD - Right Eye ________________

Vertical CD - Left Eye ________________

Horizontal CD - Right Eye ________________

Horizontal CD - Left Eye ________________

GCC Parameters

FLV - Right Eye ________________

FLV - Left Eye ________________

GLV - Right Eye ______________________

GLV - Left Eye ______________________

OCT - Right Eye

Scan quality index - Right Eye ______________________

OCT - Left Eye

Scan quality index - Left Eye ______________________

CVF - Right Eye

Scan quality index - Right Eye ______________________

CVF - Left Eye

Scan quality index - Left Eye ______________________

Would you wish to be contacted with the results of this study? ◯ Yes ◯ No
